# Performance analysis of fiber reinforced recycled aggregate concrete at elevated temperatures using response surface methodology

**DOI:** 10.1038/s41598-025-94258-w

**Published:** 2025-04-15

**Authors:** Muhammad Saqib Khan, Muhammad Imran Khan, Rafiq M. Choudhry, Shabir Hussain Khahro, Zubair Ahmed Memon

**Affiliations:** 1 National University of Sciences and Technology (NUST), Risalpur Campus, Risalpur, 24080 Pakistan; 2 Civil Engineering Department, College of Engineering, Imam Mohammad Ibn Saud Islamic University (IMSIU), 11432 Riyadh, Saudi Arabia; 3 Department of Engineering Management, College of Engineering, Prince Sultan University, Riyadh, Saudi Arabia

**Keywords:** Sustainable concrete, Recycled aggregate concrete, Elevated temperature, Fiber reinforced recycled concrete, ANOVA analysis, Civil engineering, Environmental sciences

## Abstract

The increasing risk of fire hazards and the environmental burden of construction and demolition (C&D) waste necessitate sustainable, fire-resistant building materials. This study investigates the potential of recycled aggregate concrete (RAC) reinforced with fibers to enhance high-temperature performance while promoting waste utilization. In the first phase, concrete mixtures with 0%, 25%, and 50% recycled aggregate (labeled RAC00, RAC25, and RAC50) were evaluated at 23 °C, 300 °C, and 600 °C. RAC00 exhibited a 16% reduction in compressive strength at 300 °C and over 50% at 600 °C. While RAC25 initially showed a 10–30% decrease compared to conventional concrete at room temperature, it exhibited only a 1% strength decline at 300 °C and a 28% reduction at 600 °C, making it the most effective composition for further study. In the second phase, steel fibers (SF) and polypropylene fibers (PPF) were incorporated into RAC25, yielding substantial tensile strength improvements: RAC25-SF increased by 5.6% at 23 °C, 24.8% at 300 °C, and 28.3% at 600 °C. RAC25-PPF showed a 12.5% increase at 23 °C but declined at higher temperatures, with a 9.9% decrease at 300 °C and 32.9% at 600 °C. SF enhanced strength across all temperatures, while PPF reduced performance above 200 °C. Fiber additions improved ductility, toughness, and moisture retention, mitigating crack propagation under heat. Statistical modeling using ANOVA and response surface methodology (RSM) confirmed high model validity (R^2^ > 0.80). The study concludes that RAC25 with steel fibers offers a sustainable, heat-resistant construction material, addressing both fire resilience and C&D waste challenges.

## Introduction

The construction industry generates a substantial amount of waste, with concrete waste forming a significant part of this construction and demolition waste (CDW). This waste, if not properly managed, contributes to environmental degradation due to its large volume and non-biodegradable nature. Globally, more than 3 billion tons of CDW accumulate annually, posing both an environmental challenge and a resource opportunity^1–3^. China, India, and the United States produce more than 2 billion tons of waste, creating a pressing need for effective waste management solutions^4,5^. Recycling concrete waste into aggregates and reusing it in new concrete production is a sustainable approach that conserves resources and mitigates the environmental impact of CDW^6^. Using recycled aggregate concrete (RAC) is a significant advancement in the worldwide construction industry. Recycled aggregates are used in the production of RAC, lowering the demand for virgin resources and aiding in managing debris from building and demolition. Some countries do not have sufficient concrete ingredients and have to import them or use the sea floor as a mineral source of aggregates^7^. RAC has thus emerged as a key development in sustainable construction, reducing the demand for virgin materials and providing a solution for CDW management^8^.

However, RAC typically exhibits lower quality than natural aggregate concrete (NAC), largely due to the residual mortar attached to recycled aggregates^9–11^. This residual mortar introduces additional Interfacial Transition Zones (ITZs) in the RAC matrix, which are more porous than the aggregates themselves, resulting in increased water absorption, reduced density, and diminished mechanical properties^12,13^. Specifically, RAC’s compressive strength and durability are often compromised compared to NAC, as the presence of microcracks and residual mortar weakens the aggregate-mortar bond, particularly under high-stress conditions^14,15^. To address these limitations, there is an urgent need to explore strategies that can enhance the mechanical properties of RAC, making it comparable to NAC in strength and durability.

Research on RAC has shown promising results, though it generally has lower performance compared to NAC due to the residual mortar on recycled aggregates^16^. Found that RAC’s compressive strength was around 10–20% lower than NAC, but it still met standards for non-structural applications due to its greater water absorption and porosity in the interfacial transition zone (ITZ)^17,18^. Observed that RAC with supplementary cementitious materials (SCMs) like fly ash could achieve strength levels nearly comparable to NAC, with permeability reduced by up to 25%, which helps mitigate durability issues^19^. Conducted a comprehensive review, noting that RAC strength typically ranges 10–25% lower than NAC, but treatments such as pre-soaking or using SCMs improved the durability and made RAC suitable for structural use^17^. Reported that RAC had 15–30% lower resistance to chloride ion penetration, though adding fly ash as a partial cement replacement decreased chloride ingress by 30%, making RAC feasible for specific applications, especially in non-exposed structural elements in marine environments.

The performance of concrete, including RAC, under elevated temperatures has been widely studied, as high temperatures can significantly impact concrete’s structural integrity. Elevated temperatures lead to thermal expansion, spalling, and loss of strength due to the breakdown of hydrated cement compounds^20^. For RAC, these effects are often exacerbated due to the microstructural deficiencies associated with recycled aggregates^21–23^. Knaack and Kurama^24^ found that RAC retained only 60% of its initial compressive strength after exposure to 600 °C, with increased susceptibility to spalling and cracking^25^. Demonstrated that RAC’s strength deteriorates more rapidly at temperatures above 400 °C compared to NAC, primarily due to the increased vapor pressure and cracking in residual mortar. High temperatures possess the ability to severely impact normal concrete’s structural integrity, resulting in problems including spalling, lowered compressive strength, and higher permeability^26–28^. The strength of recycled aggregate concrete decreases with higher exposure temperature and longer exposure time. The deterioration of concrete strength at high temperatures is due to vapor pressure and crack formation^29–32^. According to a study conducted by Ahmed and Lim^33^ the RAC’s thermal performance is on the same level as conventional concrete in terms of its thermal properties. According to the study, even after being exposed to temperatures as high as 600 °C, RAC was able to retain up to 60% of its initial compressive strength and structural integrity. Similar to this, a research study has shown that steel fiber-reinforced RAC had compressive strengths of up to 35 MPa and improved residual strength properties by 10–15% compared to non-fiber-reinforced RAC^34,35^.

To overcome these challenges, researchers have investigated the addition of fibers to enhance the mechanical and thermal performance of RAC. Fibers, such as steel and polypropylene, have shown promising results in reinforcing RAC by improving its strength, ductility, and thermal stability^36^. Interestingly, these investigations highlight how crucial fiber type and dosage are to maximizing RAC’s mechanical and thermal resistance. The compressive strength of cylindrical specimens is boosted by 2.38% with 50 mm crimped round steel fibers coated with 1% copper and an aspect ratio of 54.85^37^. Studies have concluded that the use of steel fibers to natural aggregate concrete with aspect ratios of 15, 25, and 35 and volume percentage fraction of fibers of 0.5, 1, and 1.5 resulted in a slight increase in ultimate strength. It turned out that 1% of the fiber volume percentage was ideal for improving strength and ductility^38^.

Despite these advances, there is limited research on the combined effect of steel and polypropylene fibers on RAC’s behavior at elevated temperatures. Additionally, most studies on fiber-reinforced RAC do not apply robust statistical methodologies to optimize the mix design and quantify the interactions between fibers and recycled aggregates in high-temperature conditions. This study addresses these gaps by utilizing Response Surface Methodology (RSM) to optimize the mix composition of RAC with fibers, aiming to achieve a balance between compressive strength, thermal stability, and sustainability. RSM, combined with Analysis of Variance (ANOVA), allows for a systematic evaluation of the effects of fiber type and dosage on RAC’s properties, ensuring that the composition is optimized for both mechanical performance and resource efficiency^39^.

The primary objective of this research is to evaluate the compressive and tensile performance of fiber-reinforced RAC under high temperatures (23 °C, 300 °C, and 600 °C) and to determine its suitability for use in fire-prone environments. This study contributes to sustainable construction by proposing an optimized fiber-reinforced RAC that retains sufficient strength and durability at elevated temperatures, thus addressing the environmental and resource challenges posed by CDW. The novelty of this research lies in its comprehensive approach to optimizing RAC composition through RSM, integrating steel and polypropylene fibers to achieve improved performance at elevated temperatures, and providing a statistically validated model for RAC mix design. This work offers new insights into the sustainable use of recycled aggregates and fiber-reinforced RAC in structural applications exposed to extreme conditions.

## Materials and methods

This section discusses the materials that were used in the research work and the different experimental procedures and methods applied to it.

### Materials

#### Cement

Ordinary Portland Cement (OPC) with a density of 3140 kg/m^3^ was used as a binder. The cement complies with^40^, *Standard Specification for Portland Cement*. The physical properties of OPC are listed in Table 1.

**Table 1:** Physical properties of cement.

Items	Properties
Specific gravity	3.14
Initial setting (min)	146^41^
Final setting (min)	232^41^
Consistency (%)	28^42^
Blaine fineness (m^2^/kg)	301
Average particle diameter (µm)	< 31^43^
28 days compressive strength (MPa)	45.70^40^

#### Aggregates

**Fig. 1:**
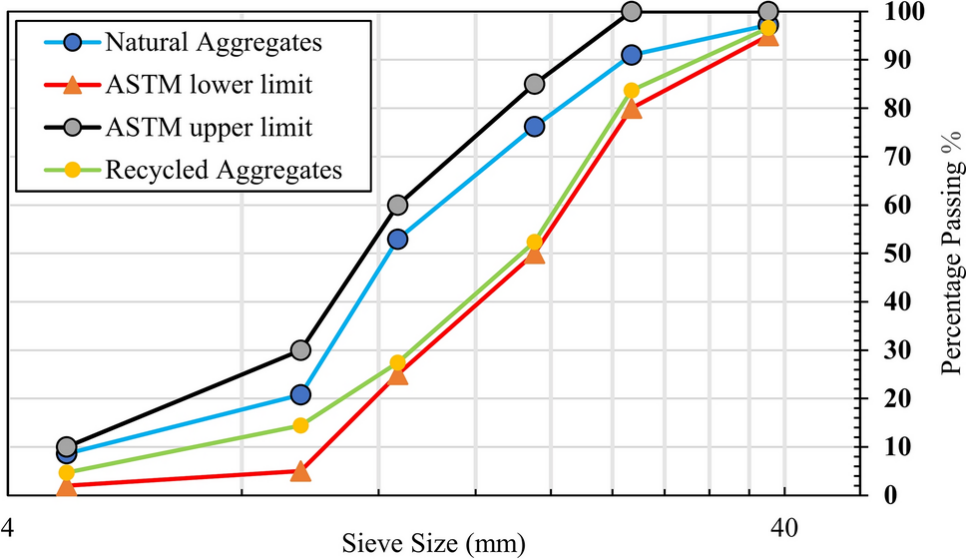
Gradation curve of aggregates.

Sand from a local source with fineness modulus 2.62 was used as fine aggregate. Normal weight crushed mineral rock was used as a coarse aggregate. Recycled aggregates were manufactured by crushing abandoned tested concrete cylinders at the concrete laboratory of National University of Sciences and Technology (NUST) Islamabad, Pakistan. Twenty-four millimeters was the maximum size of recycled aggregates and natural aggregates. Two fractions of recycled coarse aggregates were formed. The first fraction sizes were (5–15) mm and the other had (15–24) mm particle size. They were mixed in 3:2. This was done considering the idea that the smaller coarse aggregate in rich mixes increases mechanical strength of concrete by decreasing the interfacial transition zone^44^. Standard tests were carried out to determine the physical properties of natural and recycled concrete aggregates. The summary of these tests is presented in Table 2. Sieve analysis was carried out for both aggregates as per^45^, *Standard Specification for Concrete Aggregates*. Figure 1 shows the gradation of natural and recycled aggregates.

**Table 2:** Properties of aggregates.

Physical properties	Natural coarse	Recycled coarse	Fine
	Aggregates	Aggregates	Aggregates
Specific gravity	2.55	2.49	2.41
Absorption, %	1.39	5.70	1.21
Bulk density, kg/m^3^	1729.10	1406.90	1810.40
Abrasion value, %	14.60	24.07	–
Fineness modulus	2.14	2.39	2.62

#### Chemical admixture

The chemical admixture based on polycarboxylates was used to produce concrete mixes with enlarged slump retention, high water minimization, and high strength development. Due to recycled aggregate’s high-water absorption, a significant water reduction was necessary. In this work, Sika® ViscoCrete-3110 was used as a superplasticizer to obtain the best possible concrete mixture with the desired properties. Sika®ViscoCrete-3110 adapts to the^46^ requirements.

#### Steel and polypropylene fibers

**Fig. 2:**
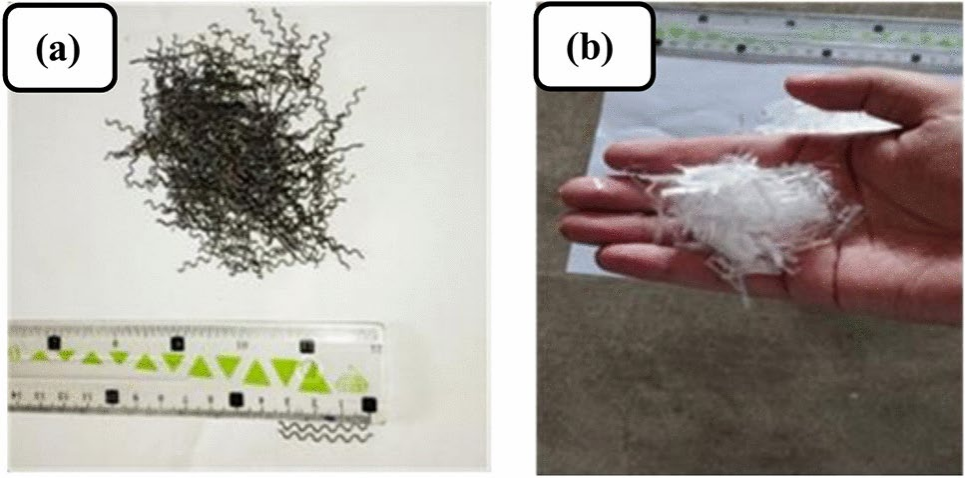
(**a**) Steel fibers, (**b**) polypropylene fibers.

This research work utilized steel fibers (Fig. 2a) crimped into round shapes, which had a melting temperature of 1500 °C, an aspect ratio of 33, and a tensile strength of 600 MPa. Additionally, Polypropylene fibers (Fig. 2b) with a fibrillated structure and a cut size of 20 mm were used. These polypropylene fibers had a specific gravity of 345 kg/m^3^. Steel fibers were chosen for their high tensile strength and elastic modulus, which enhance crack control, energy absorption, and post-crack toughness. These properties are crucial for RAC, where internal micro-cracks are more likely. Additionally, steel fibers improve residual strength at elevated temperatures, making them ideal for fire-resistant applications. Polypropylene fibers help reduce plastic shrinkage cracking, improve impact resistance, and prevent explosive spalling under fire conditions. As polypropylene fibers melt at high temperatures, they create micro-channels that release internal vapor pressure, enhancing fire resistance. Together, these fibers contribute to improving the structural resilience and fire resistance of RAC, addressing sustainability and durability in construction. The properties of the fibers are shown in Table 3.

**Table 3:** Properties of fibers.

Property	Steel fibers	Polypropylene fibers
Type	Crimped round steel fibers	Fibrillated polypropylene fibers
Material	Mild steel	Polypropylene
Manufacturer	–	Matrixx Duracrete, Karachi, Pakistan
Density (g/cm^3^)	7.85	0.91 (solid state), 0.85 (liquid state)
Length (mm)	33	20
Diameter (mm)	1.0	–
Aspect ratio (L/D)	33	–
Tensile strength (MPa)	600	–
Melting temperature (°C)	1500	171
Temperature at vaporization (°C)	–	341
Burning temperature (°C)	–	460
Thermal conductivity (W/m K)	–	0.15
Thickness (µm)	–	50
Width (µm)	–	150

## Experimental program

An experimental study was conducted to investigate the performance of recycled aggregate concrete (RAC) with fibers at moderate and elevated temperatures. The study was conducted in two phases. In the first phase, the mechanical and physical properties of RAC (recycled aggregate concrete) with 25% and 50% recycled aggregate, replaced by volume, were examined and compared with normal aggregate concrete (RAC00) regarding high-temperature behavior. Natural aggregate concrete (NAC) refers to control specimen with no inclusion of recycled aggregate (RAC00). The RAC mixtures were labeled as RAC25 and RAC50, representing the volume percentage of recycled aggregates used in replacement.

In the second phase, a standard concrete mixture incorporating recycled aggregates was established as the baseline. Steel fibers (1% by volume of concrete) and polypropylene fibers (0.8% by volume of concrete) were introduced into separate batches to investigate their contributions to the mechanical and thermal properties of recycled aggregate concrete (RAC).

Studies such as those by^47–49^ have demonstrated the benefits of steel fiber reinforcement in improving the tensile strength and impact resistance of concrete under mechanical and thermal stress. Recent research by^50–53^ corroborates the role of polypropylene fibers in enhancing the durability and fire resistance of fiber-reinforced concrete mixtures.

The chosen fiber volumes of 1% for steel fibers and 0.8% for polypropylene fibers are consistent with those recommended in the literature for optimal performance without compromising workability or inducing fiber balling. These dosages have been validated in studies such as^54–56^, ensuring that the mix retains its constructability while achieving the intended mechanical and thermal benefits.

**Fig. 3:**
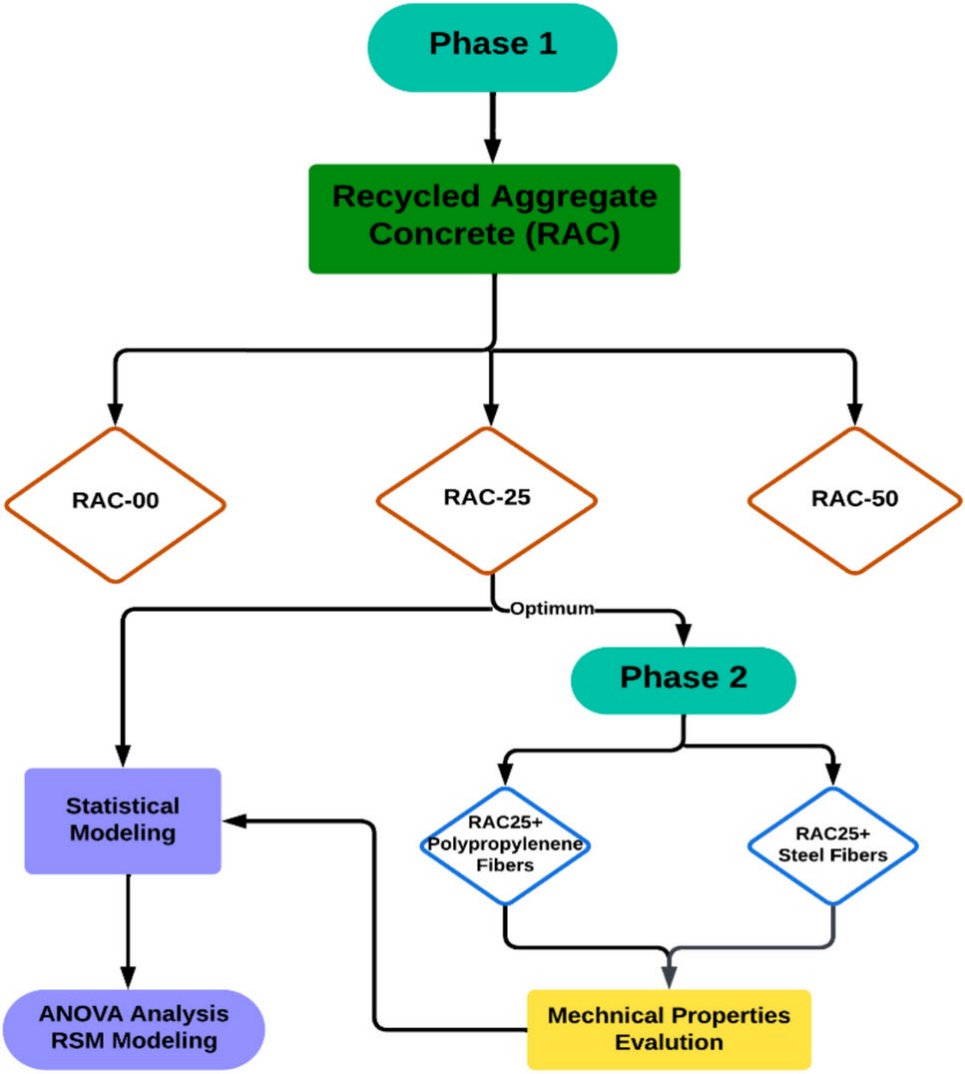
Schematic diagram of research experimental methodology.

Following the ACI mixture design^57^, the mixture proportion was decided based on laboratory trials. The detail of the mixture proportions and laboratory conditions is given in Table 4. NAC was used as a control specimen. The research schematic diagram is shown in Fig. 3. The study aimed to compare and draw conclusive research from these comparative analyses.

**Table 4:** Trail mix proportions.

Components	NAC	RAC 25% + 75% NAC	RAC 50% + 50% NAC
Cement (kg/m^3)^	279	279	279
Recycled aggregate (kg/m^3^)	0	278	555
Water content (kg/m^3^)	134	134	134
w/c Ratio	0.48	0.48	0.48
Coarse aggregate (kg/m^3^)	1197	918	642
Fine aggregate (kg/m^3^)	790	790	790
Super plasticizer (kg/m^3^)	2.78	2.78	2.78
Steel fibers (% by volume)	–	1	–
Polypropylene fibers (% by volume)	–	0.8	–

### Specimen casting and curing

Recycled aggregates were first oven-dried and then soaked in water for 24 h to account for their high-water absorption. They were thoroughly pre-wetted before mixing and allowed to reach a saturated surface dry (SSD) condition for at least 24 h, following standard procedures. During this period, periodic checks were conducted to measure the surface moisture of the aggregates, ensuring they had reached the SSD state. The amount of absorbed water was incorporated into the total mixing water to maintain consistency in the mix design^58^. A vertical drum mixer was used to combine all proportioned ingredients. Initially, coarse aggregates were added to the mixer and blended for one minute. Recycled aggregates and fine aggregates were then introduced, followed by an additional two minutes of mixing. Subsequently, ordinary Portland cement (OPC) was added along with 50% of the total mixing water to ensure even distribution. Once thorough mixing was achieved, the remaining 50% of the mixing water—pre-mixed with the superplasticizer—was gradually introduced to improve workability. This procedure was followed consistently for all recycled aggregate concrete (RAC) combinations in Phase 1.

For fiber-reinforced mixes, steel fibers (1% by volume) and polypropylene fibers (0.8% by volume) were evenly distributed into the dry mix before water was added, ensuring uniform dispersion and preventing fiber clumping. Based on the findings from Phase 1, the optimum percentage of recycled aggregates was incorporated into the fiber-reinforced concrete. After thoroughly mixing all ingredients with the fibers, the remaining mixing water was gradually added to achieve a homogenous mixture.

Cylindrical specimens were cast to evaluate the compressive strength, tensile strength, elastic modulus, and fracture properties of the studied concretes. After casting, the specimens were kept in molds for 24 h before de-molding and subsequently cured in a water tank at an average temperature of 23 °C for 28 days. Five concrete batches were prepared for each temperature variation, resulting in a total of 90 concrete cylinders.

### Tests description

Uniaxial compressive tests were performed using a servo-hydraulic universal testing machine with a capacity of 1200 kN, on all the mixes after curing for 28 days at room temperature. A split cylinder tensile strength test was conducted on the mixtures at 28 days to determine their tensile capacity, following the specified standard procedures as per^59^.

### Fire loading characteristics

**Fig. 4:**
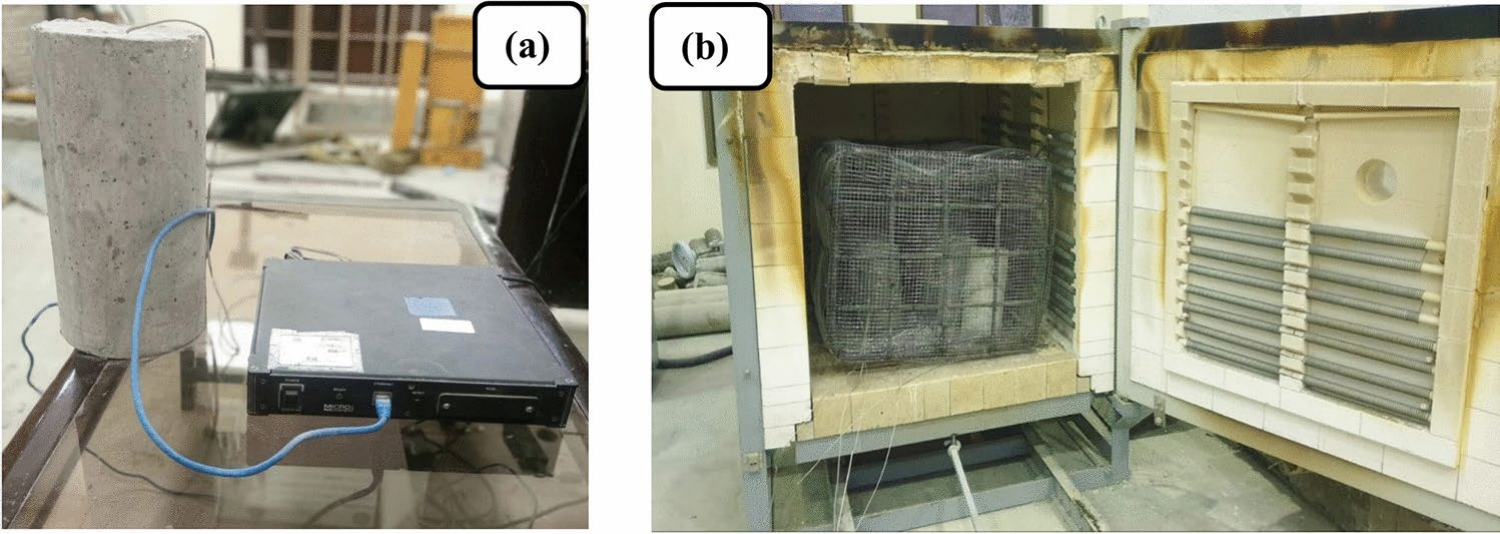
(**a**) K-type thermocouples drilled inside and on the surface of the specimen along with a data logger to record real-time temperature variations. (**b**) Heat furnace along with cage to avoid any sort of spalling of concrete.

The test results of specimens are primarily dependent upon the characteristics of applied fire loading. There are two essential fire loading characteristics on which the results are dependent. These characteristics are the heating rate and the target temperature. In the study, temperatures used to test the mechanical and thermal properties of concrete were 23 °C (room temperature), 300 °C, and 600 °C. To prevent significant tensile stresses that could lead to high-temperature differentials and spalling, a slow heating rate of 5 °C/min was employed during the testing. Heating a concrete sample in an electric furnace causes the temperature inside the furnace to rise at a certain rate. However, the temperature increase of the cylinder’s surface and core is slower than that of the furnace air. As a result, the furnace air temperature reaches the desired level much faster than the cylinder’s temperature. The heat furnace is shown in Fig. 4a.

Two type-K thermocouples were embedded in a concrete cylinder for temperature measurement during heating as shown in Fig. 4b. One was placed in the core and the other was surface-mounted. The temperature data was recorded and plotted over time using a data logger. The cylinder was heated to 300 and 600 °C for two temperature readings. It took 2 h for the core of the cylinder to reach 300 °C temperature, so a hold time of 2 h was given for all mixes. Similarly, it took 3.5 h for the core to reach 600 °C temperature, so a hold time of 3.5 h was set for the specimens.

### RSM-based design of experiment

RSM is a statistical technique that is convenient to use and is frequently applied to design, analysis, optimization, and validation of experimental data. The concrete industry has made extensive use of it for experiment design, statistical model development, and correlation analysis between causes (independent variables) and responses (dependent variables). The most widely utilized method for designing experiments and determining the components for analysis in this study was central composite design (CCD). For this^60^, Design Expert 13.0 software was utilized. Compressive strength and tensile strength were selected as responses in the current investigation, while temperature and RAC quantities were taken into consideration as influencing factors. RSM was used to statistically examine the mechanical parameters in terms of compressive and tensile strength. Similarly, linear and quadratic polynomial equation models were proposed to forecast the strength characteristics of concrete depending on the model’s importance. The answers were predicted using a second-degree polynomial equation, and Eq. 1 displays the generalized equation for the anticipated model that was applied.

1 $$\begin{array}{*{20}c} {Y = C + A_{1} \left( {X_{1} } \right) + A_{2} \left( {X_{2} } \right) + A_{3} \left( {X_{1}^{2} } \right) + A_{4} \left( {X_{2}^{2} } \right) + A_{5} \left( {X_{1} *X_{2} } \right)} \\ \end{array}$$

where X_1_ and X_2_ are the independent variables (i.e., temperature and %RAC), C is the interception constant, A_1_ through A_5_ are the equation’s coefficients, and Y is the response derived from the experimental data (i.e., compressive strength, tensile strength). Following the selection of components at low and high levels (as shown in Table 5), a design for 54 runs of the experiments was produced. To improve reliability throughout the design and analysis of the experiment, the major points were replicated five times. The design expert software’s relevant slots were filled using the experimental data of compressive and tensile strength. The %RAC and Temperature components were then analyzed, modeled, and optimized.

**Table 5:** Design of factors and relevant code in RSM.

Factors	Units	Code		Levels	
RAC	%	X1	00	25	50
Temperature	°C	X2	25	300	600

To evaluate the understanding of the model’s performance, the ANOVA was conducted. The regression models’ suitability was assessed using the ANOVA’s *F*-value, *p*-value, and lack-of-fit metrics. Comparably, the design expert creates a 3D model graph to show how two elements affect the chosen answer using the suggested model.

## Results and discussions

### Compressive strength

**Fig. 5:**
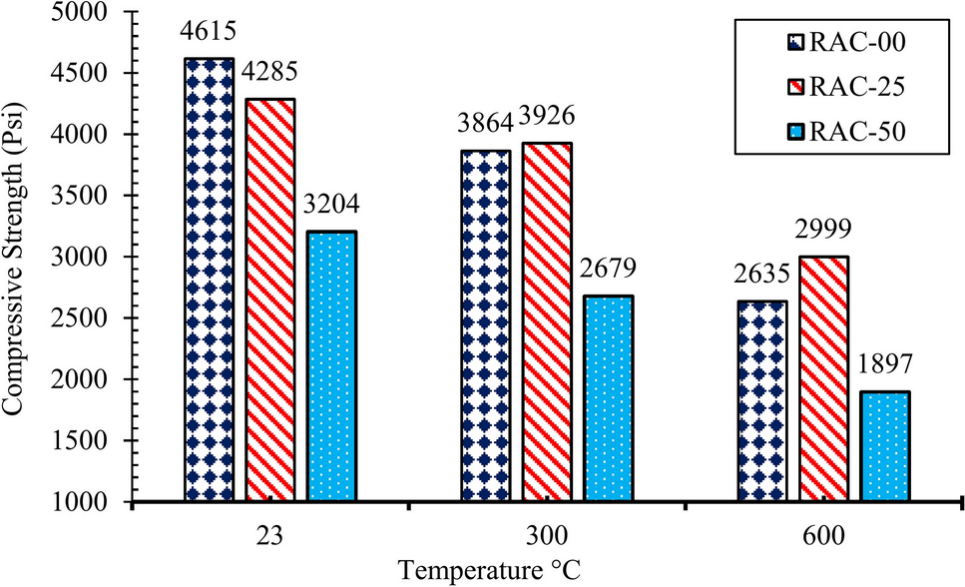
Compressive strength of RAC at different temperatures.

The average compressive strength of all the concrete mixes is shown in Fig. 5. NAC 28-day strength achieved at room temperature was about 4615 psi. Upon heating the samples to 300 °C, a 16% strength decrease was noted which further declined sharply to more than 50% of residual strength at 600 °C. As compared to NAC, compressive strength of concrete with 25% and 50% recycled aggregates decreases by 10–30% at 23 °C. However, at 300 °C RAC25 exhibited a significantly lower decline in strength, with only a 1% reduction compared to ambient temperature. The same was observed for RAC50. At 600 °C, RAC25 showed a 28% loss of strength, and RAC50 exhibited a 60% decline. This shows that concrete specimens with recycled aggregates were less prone at high temperatures. Test results of RAC25 were nearer to NAC at all temperature ranges. Therefore, RAC25 was selected as a new baseline before the addition of fibers to concrete.

**Fig. 6:**
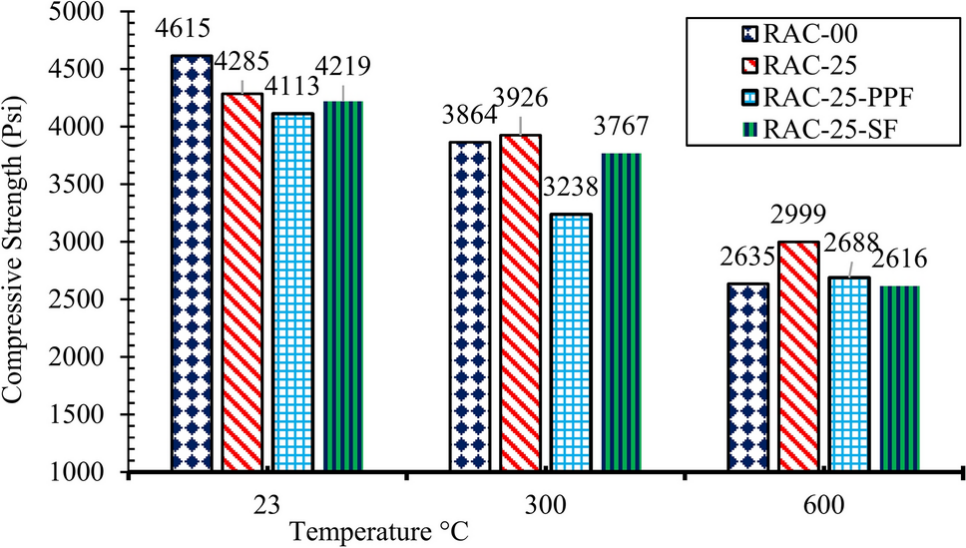
Compressive strength of RAC with fibers at different temperatures.

Prior research^61,62^ indicated that 1% steel fibers by volume and 0.8% by volume dose of polypropylene fibers can be added to the normal concrete. In this work, respective quantities of SF and PPF were added as an additive to the RAC. The test results for steel fibers in concrete showed that the compressive strength at all temperature ranges was comparable to RAC25 (Fig. 6). This indicates that steel fibers effectively contributed to the concrete’s compressive strength, even under elevated temperatures. However, a slight decrease in strength was observed at higher temperatures due to thermal stresses generated within the steel fibers and the surrounding matrix. This phenomenon is likely caused by differential expansion between the fibers and concrete, resulting in internal stresses that reduce strength. These findings are consistent with existing literature, where steel fibers have been shown to maintain concrete strength at elevated temperatures but can experience minor reductions due to thermal effects^49^. This is consistent with the findings of previous studies, such as those by^63,64^, observed that RAC generally exhibits lower compressive strength due to the weaker bond between recycled aggregates and cement paste. However, at 300 °C, RAC25 demonstrated greater compressive strength than NAC, a finding that aligns with studies by^65^, who reported that the presence of recycled aggregates could help mitigate the adverse effects of elevated temperatures on concrete by promoting more uniform temperature distribution. Steel fibers thus help in maintaining the structural integrity of RAC under heat, though their contribution slightly diminishes with temperature rise.

In contrast, polypropylene fibers showed a noticeable reduction in compressive strength compared to RAC25 across all temperature levels. At ambient temperature, the strength was 3% lower, increasing to 10% lower at 300 °C, with a more significant decline at 600 °C. This decline is primarily due to the melting of polypropylene fibers at 171 °C, which significantly weakens their contribution to the concrete’s strength at elevated temperatures. As the fibers melt, voids form in the matrix, weakening the compressive strength. Polypropylene fibers, therefore, contribute less to RAC’s compressive strength at elevated temperatures, emphasizing the need to carefully select fiber types based on the intended thermal conditions of the concrete.

### Splitting tensile strength

**Fig. 7:**
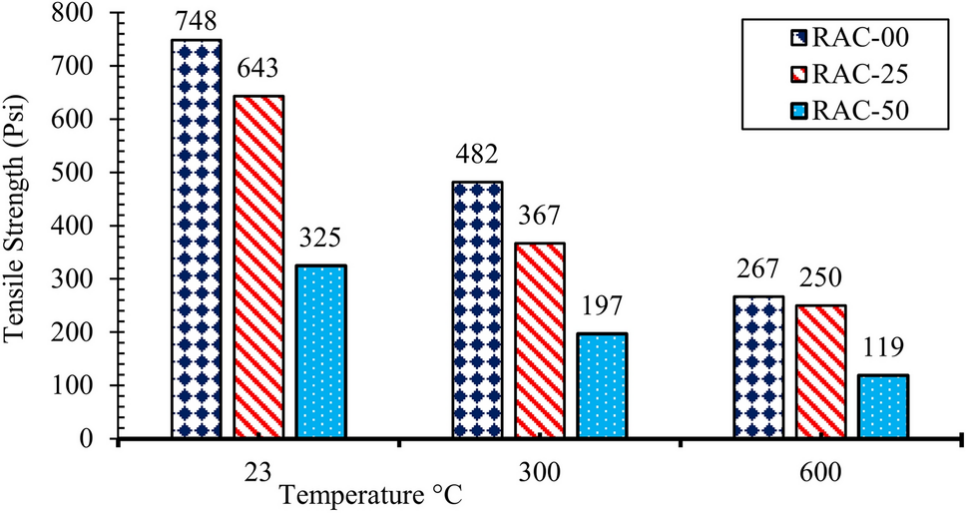
Tensile strength of RAC at different temperatures.

**Fig. 8:**
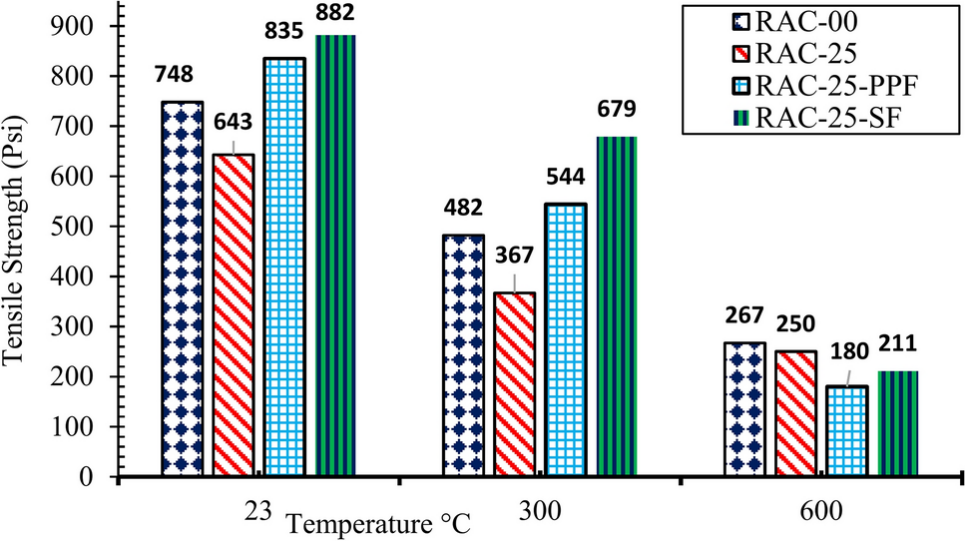
Tensile strength of RAC with fibers at different temperatures.

The mean values of splitting tensile strengths are plotted in Figs. 7 and 8. NAC tensile strength drops by 40% at 300 °C and at 600 °C NAC specimens retained only 35% of their original tensile strength. Tests on concrete with 25% and 50% recycled aggregates showed that the tensile strength decreased rapidly at higher temperatures. Contrary to this, the addition of steel fibers significantly enhanced the tensile strength of recycled aggregate concrete (RAC), surpassing that of normal aggregate concrete (NAC). This improvement is primarily attributed to the fibers’ ability to bridge cracks and distribute stress, which enhances the overall structural integrity of the concrete. This aligns with the findings of^66^, who showed that RAC tends to maintain tensile strength better than NAC at high temperatures due to the thermal expansion properties of recycled aggregates. At 300 °C, a slight decrease in tensile strength was observed, which can be attributed to the thermal stresses and chemical changes occurring within the bond between the fibers and the concrete matrix. Despite this reduction, no rapid decline was noted, indicating that steel fibers contribute to maintaining the tensile strength even at elevated temperatures.

On the other hand, polypropylene fibers showed acceptable tensile strength at room temperature, outperforming RAC25. However, the tensile strength of the mix with polypropylene fibers decreased significantly at elevated temperatures. This is because polypropylene fibers melt at 171 °C, which compromises their reinforcing role and results in a rapid decline in tensile strength. The results suggest that while polypropylene fibers can improve tensile strength at ambient temperature, their performance deteriorates at elevated temperatures due to their melting behavior.

### Stress–strain curve at different temperatures

**Fig. 9:**
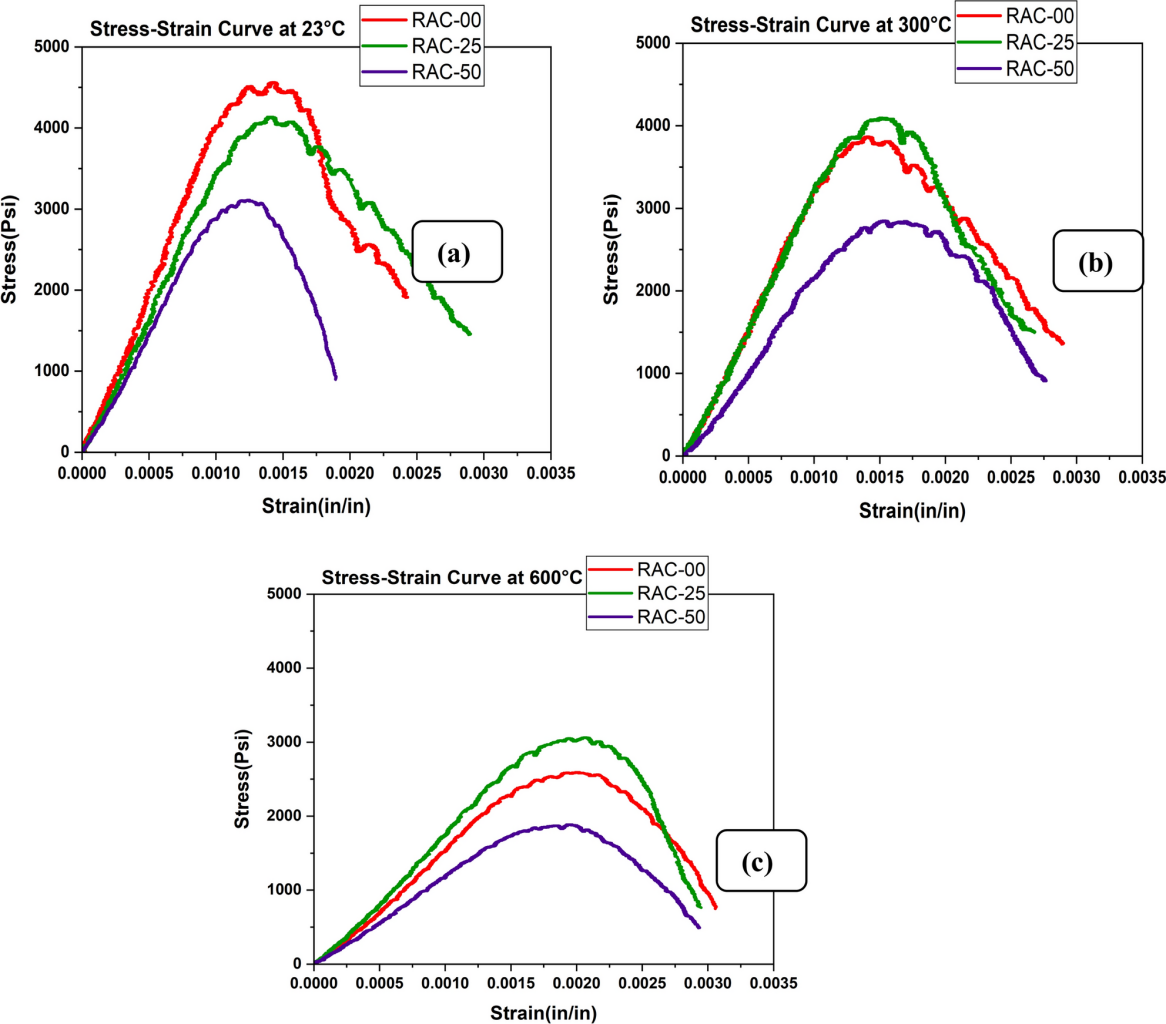
Stress–strain curve of RAC at (**a**) 23°C, (**b**) 300°C and (**c**) 600°C.

The stress–strain response of the concrete mixes under varying temperatures reveals significant effects of fiber addition on both strength and ductility (Fig. 9a–c). At room temperature, normal aggregate concrete (NAC) exhibited higher ultimate strength and more ductile behavior than both RAC25 and RAC50. However, as the temperature increased to 300 °C, RAC25 demonstrated a higher residual strength than NAC, while RAC50 showed a milder slope and a decrease in ultimate strength. Despite these variations, the peak strain values for all mixes increased to around 0.3%, suggesting that all mixes, including RAC50, became more ductile at higher temperatures, as shown in Fig. 9.

**Fig. 10:**
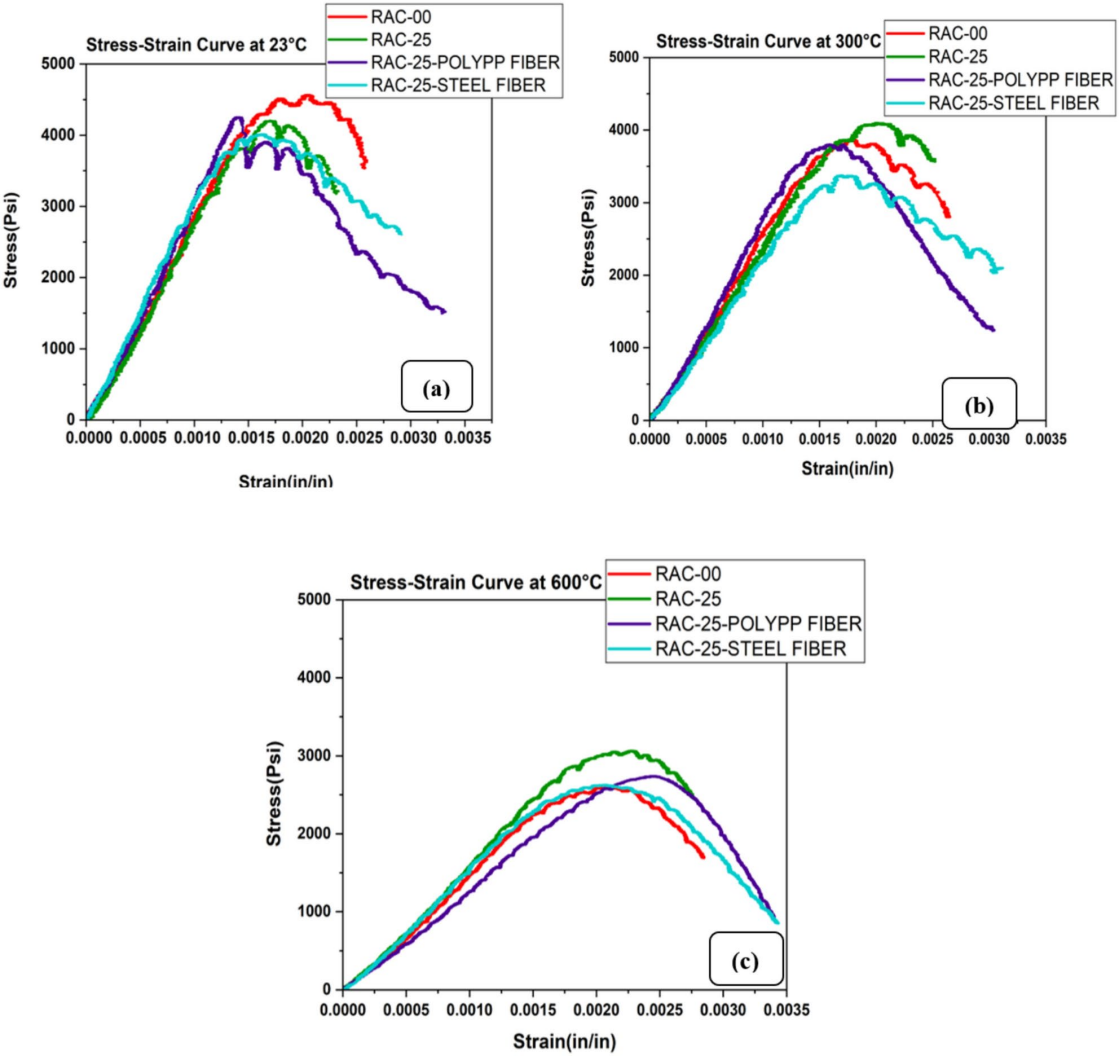
Stress–strain curve of RAC and fibers at (**a**) 23 °C, (**b**) 300 °C and (**c**) 600 °C.

The addition of steel fibers to the RAC25 mix resulted in improved ductility, with a maximum strain of 0.45%, which was higher than the normal ductility of concrete (as shown in Fig. 10a–c). While steel fibers did not significantly increase the ultimate strength at room temperature, they effectively enhanced the concrete’s resistance to crack propagation and widening, thus improving ductility. As illustrated in Fig. 10a, at 300 °C, steel fibers slightly increased ductility, although they did cause a slight decrease in ultimate strength due to thermal stresses and bond behavior changes. At 600 °C, steel fibers maintained the highest strain limit of 0.5%, proving their ability to sustain high ductility at elevated temperatures, as seen in Fig. 10c. In contrast, polypropylene fibers demonstrated a maximum strain of 0.5% at room temperature, which was higher than steel fibers, despite not contributing to increased strength. This behavior was similar to the findings of^67^, who noted that while polypropylene fibers help mitigate cracking by releasing thermal stresses, their tensile and strain capacities diminish under high-temperature conditions. However, their performance diminished at higher temperatures: at 300 °C, the strain limit decreased due to the fibers’ melting point (171 °C), although their fracture strength remained high. At 600 °C, the strain limit for RAC with polypropylene fibers was 0.33%, and their fracture strength remained notable, but steel fibers still showed superior performance in maintaining ductility, as shown in Fig. 10c.

These findings highlight the distinct roles of steel and polypropylene fibers in improving the ductility of recycled aggregate concrete (RAC), with steel fibers providing more consistent performance at elevated temperatures, while polypropylene fibers primarily enhance ductility at lower temperatures but lose effectiveness in high heat.

### Elastic modulus

Static modulus of elasticity according to (ASTMC469/C469M-14 2014) was calculated from the data from the above-shown stress–strain plots. Usually, the rise in the temperature results in the reduction of the Elastic modulus (ET). In concrete, the loss of modulus of elasticity depends on several parameters like type of aggregates, water cement ratio, exposure temperatures and microstructure of the concrete.

The loss of elastic modulus in the initial rise in temperature is ascribed to the transformation of cement hydrates which causes change microstructure and results in rising in the porosity and then eventually the loss of water (chemically bound) produced due to this reaction. The elastic modulus of NAC, RAC25 and RAC50 at room temperature, 300 °C and 600 °C and their RAC25 combination with SF and PPF is shown in Tables 6 and 7 respectively.

**Table 6:** Elastic Modulus of trail mix.

	Room temperature	300 °C temperature	600 °C temperature
	Elastic modulus	Elastic modulus	Elastic modulus
	(Chord)	(Chord)	(Chord)
	Psi × 10^6^	Psi × 10^6^	Psi × 10^6^
NAC	4.83	3.13	1.43
RAC25	3.87	3.22	1.69
RAC50	2.56	2.03	1.17

**Table 7:** Elastic modulus of optimum concrete mix.

	Room temperature	300 °C temperature	600 °C temperature
	Elastic modulus	Elastic modulus	Elastic modulus
	(Chord)	(Chord)	(Chord)
	Psi × 10^6^	Psi × 10^6^	Psi × 10^6^
RAC25 + SF	1.95	1.57	1.12
RAC25 + PPF	1.90	1.39	1.25

At room temperature, NAC has the highest elastic modulus (4.83 × 10^6^ psi), followed by RAC25 (3.87 × 10^6^ psi), and RAC50 (2.56 × 10^6^ psi), with fibers slightly lowering the modulus. At 300 °C, the modulus drops for all mixes, with RAC25 + SF retaining more stiffness (1.57 × 10^6^ psi) than RAC25 + PPF (1.39 × 10^6^ psi). At 600 °C, the modulus significantly decreases, with NAC maintaining the highest value (1.43 × 10^6^ psi) and RAC25 + SF (1.12 × 10^6^ psi) outperforming RAC25 + PPF (1.25 × 10^6^ psi) due to the stability of steel fibers. Overall, fibers improve ductility and thermal performance, but the modulus decreases as temperature rises.

### Cracking behavior and mass loss

The study examined the formation of cracks and the weight loss of different concrete mixtures when subjected to high temperatures. In normal aggregate concrete (NAC), wider cracks appeared on the surface of concrete specimens as the temperature increased, and these cracks became larger and more spread out in recycled aggregate concrete (RAC). Conversely, concrete cylinders with added fibers showed no visible cracks at 300 °C, and only small hairline cracks were visible at 600 °C, in comparison to recycled aggregate concrete. The addition of steel fibers and polypropylene fibers also impacted the weight loss of RAC samples. At 300 °C, both types of fibers considerably reduced the weight loss of RAC25 mixes, while at 600 °C, only steel fibers reduced weight loss. This suggests that adding fibers to RAC can help reduce the amount of moisture released when exposed to high temperatures, potentially increasing its resistance to thermal stresses and cracking.

### Statistical analysis of RAC with fibers under high temperatures using RSM

Using the RSM technique, the strength properties of the RAC and the best possible RAC with fibers (steel and polypropylene fibers) were examined, optimized, and validated. The primary goal of this analysis was to assess the effects of key variables, such as temperature and recycled aggregate content, on the mechanical properties of the concrete. Responses such as elastic modulus, tensile strength, and compressive strength (28 days) were evaluated, while different percentages of RAC and temperature were selected as variables. The ANOVA approach was adopted to statistically examine the impact of these variables on responses and verify the significance of the proposed models. This section aims to provide a deeper understanding of how these variables interact and optimize the concrete mix for improved performance under various temperature conditions.

#### Statistical assessment of concrete containing different fractions of RA

RSM was utilized to statistically investigate mechanical variables, specifically with compressive and tensile strength. To assess a deeper understanding of the model’s performance in terms of compressive and tensile strength, an ANOVA analysis was conducted inside RSM^68,69^. Similarly, quadratic polynomial models were proposed to forecast the strength attributes of RAC based on the model’s importance. The generalized Eq. (1) can be modified to Eq. (2) based on the selected factors as shown below.

2 $$\begin{array}{*{20}c} {Y = C + A_{1} \left( {RAC} \right) + A_{2} \left( {Temp} \right) + A_{3} \left( {RAC^{2} } \right) + A_{4} \left( {Temp^{2} } \right) + A_{5} \left( {RAC{*}Temp} \right)} \\ \end{array}$$

where Y is the response obtained from experimental results, i.e., Compressive and Tensile strengths, Elastic Modulus where RAC and Temp are the independent parameters, C is the interception constant, and A_1_ to A_5_ are the coefficients of the equation. The model and fit statistics from the ANOVA analysis are shown in Table 8 to determine the relevance of established models. The models are significant, and there is substantial agreement between the variables and responses for all responses, as indicated by the higher *F*-values and lower *p*-values (< 0.04). Additionally, the larger R^2^ value (> 0.80) and the smaller numerical difference (< 0.2) between the adjusted and anticipated R^2^ support the significance of the established models in response prediction. The coefficient of determination, or R^2^, is a measure of the quality and degree of fitness of constructed models. The model is well-fitted and there is a large amount of agreement between the actual and projected responses, as shown by an R^2^ value of > 0.80.

**Table 8:** ANOVA analysis and model validation for recycled aggregate concrete.

Responses	Compressive strength	Tensile strength	Elastic modulus
	Psi	Psi	Psi
Standard deviation	128.94	44.81	36,853.87
Mean R^2^	0.9813	0.9516	0.9903
Predicted R^2^	0.9769	0.9401	0.9880
Adjusted R^2^	0.9700	0.9201	0.9840
Adequate precision	42.6799	27.6233	56.8083
*F*-value (model)	220.87	82.63	429.31
*p*-value (model)	< 0.0001	< 0.0001	< 0.0001
Model	Significant	Significant	Significant
Lack of fit (*F* and *p* values)	Not significant (*F* value = 6.83 *p* value = 0.0029)	Not significant (*F* value = 4.96 *p* value = 0.0111)	Not significant (*F* value = 26.03 *p* value ≤ 0.0001)
Final model type	Quadratic	Reduced quadratic	Quadratic

Moreover, a lack-of-fit analysis may be used to assess the model’s suitability and the variance in the data around the fitted model^70^. Well-fitting models are indicated by less lack-of-fit *F*-value and in-significance (*p*-value > 0.04). The ANOVA analysis shown in Table 8 demonstrates that the chosen models are highly significant, well-fitting, and useful for establishing a connection between variables and responses because of their reduced F-value and in-significance lack-of-fit error. The results clearly show that % RAC significantly influences the compressive and tensile strength of concrete. Equations (3) to (5) are the empirical equations developed based on the actual factors (RAC and Temperature) to predict the responses after model reduction. The model reduction technique was applied to remove insignificant terms.

3 $$\begin{aligned} C.S & = 4588 + 17*\left( A \right) - 1.42*\left( B \right) + 0.023* \left( {A * B} \right) - 0.9* \left( {A^{2} } \right) \\ & \quad - 0.0028 *\left( {B^{2} } \right) \\ \end{aligned}$$

4 $$\begin{aligned} T.S & = 744.3 - 8.36 *\left( A \right) - 0.911*\left( B \right) + 0.0095*\left( {A*B} \right) - 0.0057*\left( {A^{2} } \right) \\ & \quad + 0.00024*\left( {B^{2} } \right) \\ \end{aligned}$$

5 $$\begin{aligned} E.M & = 1.18858E06 - 4675.81*\left( A \right) - 2602.65*\left( B \right) + 17.94*\left( {A*B} \right) \\ & \quad - 131.12*\left( {A^{2} } \right) + 1.482*\left( {B^{2}} \right) \\ \end{aligned}$$

where C.S = Compressive Strength; T.S = Tensile Strength; E.M = Elastic Modulus; A = RAC in %; B = Temperature in °C.

**Fig. 11:**
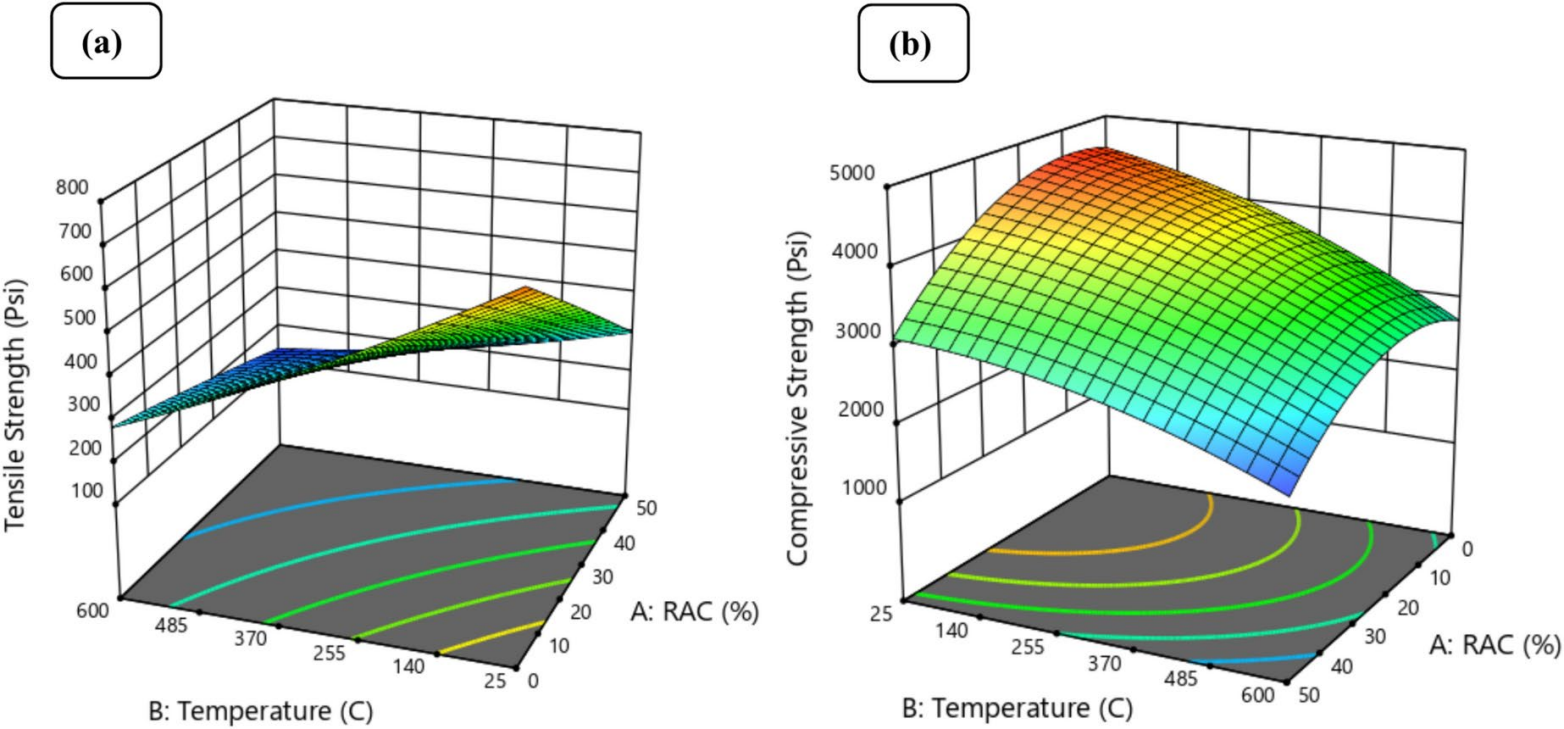
Effect of RAC and temperature on (**a**) tensile strength, (**b**) compressive strength.

Furthermore, 3-dimensional (3-D) diagrams are the most effective means of illustrating the link between the independent factors (RAC and Temp) and the related dependent variables (compressive strength, tensile strength, and elastic modulus). The three-dimensional response surface for temperature, RAC, and tensile strength is shown in Fig. 11a, and the three-dimensional response surface for temperature, RAC, and compressive strength is shown in Fig. 11b. The strength properties increase when the temperature and RAC concentration tend to decrease.

#### Statistical assessment of concrete containing RAC and fibers

The impact of adding fibers (PPF and STF) on compressive and tensile strength is covered in this section. The ANOVA analysis for the model and fit statistics to gauge the significance of well-established models are shown in Table 9. The established models indicated a strong degree of agreement between the variables and answers; the models’ lower *p*-value and higher *F*-value indicate their significance. Comparably, more appropriate frequency, better R^2^ (> 0.80), and insignificance of a lack-of-fit show that the proposed models are well-fit and noteworthy. Thus, a link between independent factors (temperature and fibers) and dependent variables (compressive and tensile strength) may be established using these models^71^. Furthermore, Table 9 describes the contribution percentages of elements that may be used to evaluate the impact of each component on the qualities of concrete. The results clearly show that temperature and fiber content have a major impact on the strength characteristics of concrete.

**Table 9:** ANOVA analysis and model validation for recycled aggregate concrete with fibers.

Responses	Compressive strength	Tensile strength	Elastic modulus
	Psi	Psi	Psi
Standard deviation	162.84	75.88	61,425.23
Mean R^2^	0.9478	0.9302	0.9452
Predicted R^2^	0.9354	0.9135	0.9321
Adjusted R^2^	0.9174	0.8889	0.9108
Adequate precision	22.5719	20.7510	27.8076
*F*-value (model)	76.25	55.95	72.41
*p*-value (model)	< 0.0001	< 0.0001	< 0.0001
Model	Significant	Significant	Significant
Lack of fit (*F* and *p* values)	Not significant (*F* value = 10.05 *p* value = 0.0004)	Not significant (*F* value = 5.75 *p* value = 0.0061)	Not significant (*F* value = 7197.11 *p* value ≤ 0.0001)
Final model type	Quadratic	2FI	Quadratic

**Fig. 12:**
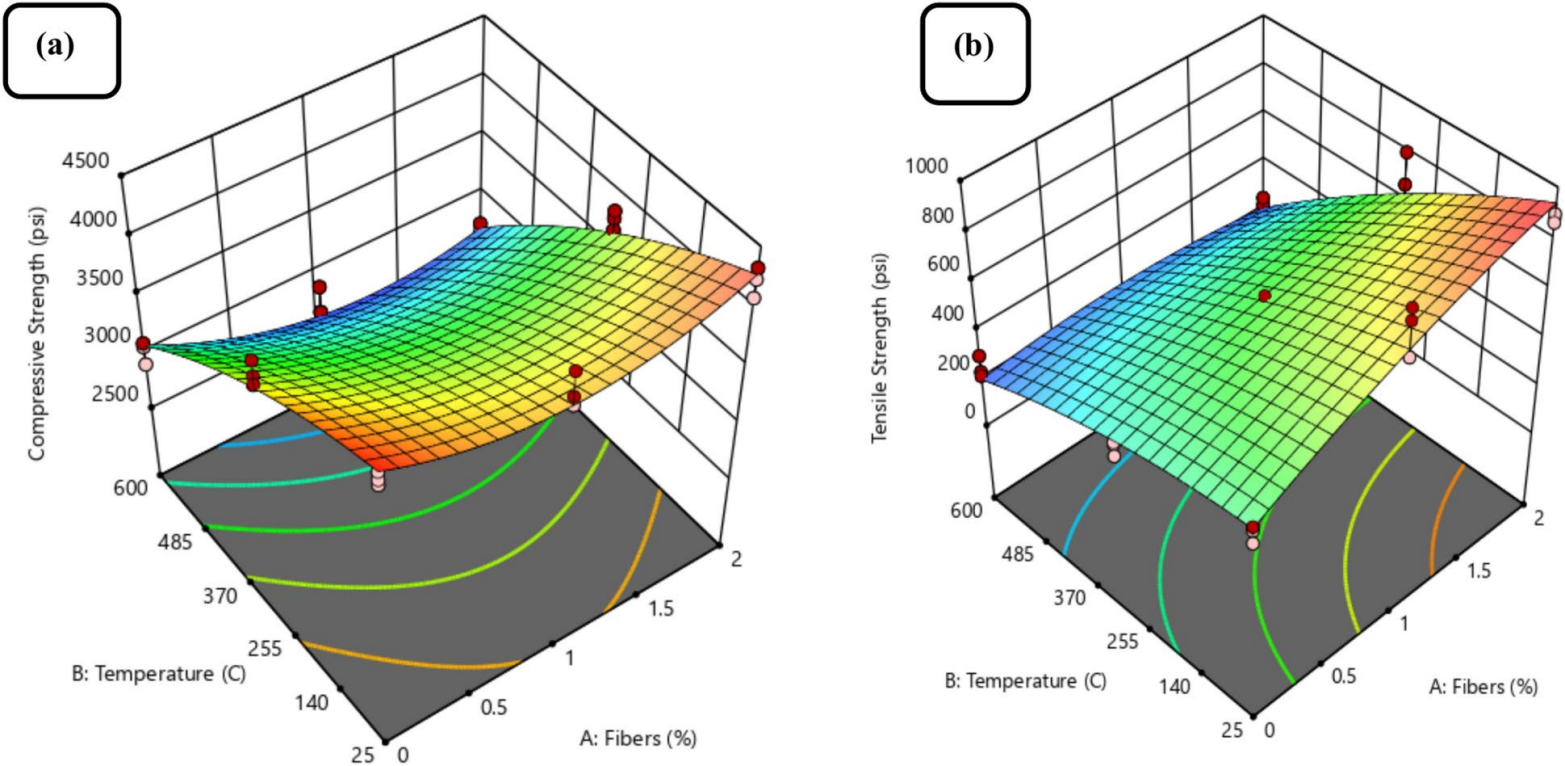
Effect of RAC with fibers and temperature on (**a**) compressive strength, (**b**) tensile strength.

The coefficients of empirical models derived from ANOVA in terms of actual factors (i.e., Fibers and Temperature) to predict Compressive and Tensile strength are presented in Eqs. (6) to (8). In addition, 3-dimensional (3-D) diagrams can also be used to best describe the relationship between factors and responses, as illustrated in Fig. 12a and b.

6 $$\begin{aligned} C.S & = 4336 - 599.4*A - 1.02*B - 0.32*\left( A *B \right) \\ & \quad + 295*A^{2} - 0.0018*B^{2} \\ \end{aligned}$$

7 $$\begin{aligned} T.S & = 487 + 326.1 * A - 0.126* B - 0.40* \left( A*B \right) \\ & \quad - 43.8191 * A^{2} - 0.00058* B^{2} \\ \end{aligned}$$

8 $$\begin{aligned} E.M & = 871566 - 549906 * A - 1171.66 * B + 245.203 * \left( A*B \right) \\ & \quad + 180434 * A^{2} + 0.252 *B^{2} \\ \end{aligned}$$

#### Multi-objective optimization and validation of modeled results

Using the RSM tool, a multi-objective optimization method was applied to get the best result. Since determining the optimal parameters depending on each person’s response is difficult. Compressive strength (28 days) and tensile strength were regarded as dependent responses in the current investigation, whereas RAC and RAC with fiber and Temperature were chosen as independent variables. The criteria for variables and responses were established following the completion of the ANOVA analysis. Seeing how fibers replace cement in the strength qualities of RAC is an intriguing observation. It can be concluded from optimization results that using 25% RAC and adding steel fibers to the concrete will significantly increase its tensile strength. Furthermore, additional experiments were conducted to validate the theoretical results from the analysis and to justify the agreement between predicted and experimental results. There is significant compatibility between predicted and experimental results with an error of < 5%.

## Conclusion

This work investigated the effect of adding steel fibers and polypropylene fibers to recycled aggregate concrete (RAC) and their performance at elevated temperatures of 300 °C and 600 °C. The following conclusions are drawn from the experimental investigation:

- At room temperature, natural aggregate concrete (NAC) exhibited the highest compressive strength compared to RAC25 and RAC50. However, at 300 °C, RAC25 demonstrated greater compressive strength than NAC. At 600 °C, all specimens experienced a significant decline in strength, retaining less than 50% of their original strength. Steel fibers did not significantly improve compressive strength but contributed to ductility and crack control, particularly under high-temperature conditions.
- Polypropylene fibers (PPF) melted at elevated temperatures (171 °C), leading to a loss of their direct contribution to compressive strength. However, their melting helped release thermal stresses, reduce spalling, and mitigate cracking, indirectly reinforcing the composite. This aligns with existing research showing that PPF improves thermal performance rather than directly enhancing compressive strength.
- The tensile strength of NAC decreased significantly as the temperature rose. In contrast, RAC25 and RAC50 exhibited a more gradual reduction in tensile strength, maintaining performance up to 600 °C. At room temperature, fibers enhanced the tensile properties of RAC. At elevated temperatures, the tensile strength of RAC with fibers approached that of NAC, highlighting their thermal resistance contribution.
- Stress–strain behavior analysis revealed that adding fibers to RAC enhanced its ductile properties and ultimate strength at elevated temperatures. RAC25 with steel fibers outperformed NAC in terms of ductility at 600 °C, while RAC25 with PPF did not perform adequately due to its low melting temperature.
- Thermal stability of RAC with steel fibers: Steel fibers significantly improved the ductility and crack resistance of RAC, even at elevated temperatures. The maximum strain for RAC25 with steel fibers increased to 0.45% at room temperature and 0.5% at 600 °C, indicating enhanced resistance to thermal cracking.
- Polypropylene fibers exhibited higher strain capacity (up to 0.5%) than steel fibers at room temperature, reflecting their ability to accommodate deformation. However, at higher temperatures, the melting of PPF reduced its strain capacity but still mitigated explosive spalling.
- The addition of fibers enhances ductility and thermal performance, but results in a reduction in elastic modulus as temperature increases, with steel fibers outperforming polypropylene fibers at elevated temperatures.
- Incorporating fibers into RAC reduced the emission of moisture at elevated temperatures, thereby minimizing thermal strains and limiting crack propagation. This effect was more pronounced with steel fibers, which enhanced the thermal stability and overall performance of RAC under high-temperature conditions.
- A statistical model using ANOVA was created to analyze the compressive strength, tensile strength, and elastic modulus with the RAC content and temperature. The models were validated and found to have high R^2^ (> 0.80) and adequate precision (> 4.0), as well as lower *p*-values and no lack-of-fit significance. This confirms that all the models are significant, well-fitted, and can be used for predicting the responses.

For future research, it is recommended to further explore the long-term durability of RAC with fiber reinforcement under cyclic heating and cooling conditions would provide a more comprehensive understanding of the fibers’ impact on thermal fatigue and crack propagation. Furthermore, conducting a more detailed study into the synergistic effects of fiber combinations, such as steel and polypropylene, on the thermal stress mitigation and crack control properties of RAC, could lead to optimized mix designs for high-temperature applications. Finally, incorporating more advanced modeling techniques and simulation tools could help predict the behavior of fiber-reinforced RAC in real-world conditions, assisting in the design of more efficient and durable concrete structures.

## Data Availability

The data that support the findings will be available on demand from the corresponding author.
